# American Dental Association

**Published:** 1886-01

**Authors:** 


### AMERICAN DENTAL ASSOCIATION.

TWENTY-FIFTH ANNUAL MEETING, HELD AT MINNEAPOLIS, MINN., AUGUST 4, 5, 6, AND 7, 1885.

REPORTED FOR THE INDEPENDENT PRACTITIONER BY "MRS. M. W. J."

(Continued from page 651, Vol. VI.)

### THURSDAY MORNING SESSION.

*Dr. Frank Abbott*—Wished to say one word with reference to the Herbst method, as he had had a little experience. The INDEPENDENT PRACTITIONER had published illustrations of his set of instruments. It was impossible to get into all cavities with straight instruments, while the proper curve would admit of doing a hundred things that could not otherwise be accomplished. The object of his new curved shapes was to get into undercuts. It is not safe to depend entirely upon the Herbst method. The rubbing process, without annealing, will not give sufficient cohesion to resist, where there is any purchase. A filling made with the electric mallet, and annealed red hot all the way through, will not break apart. He remarked that nothing had been said in the discussion, of the bridge-work of Dr. Parmly Brown. He was well pleased with the specimens he had observed, both in the hand and in the mouth. It was the finest he had yet seen, admitting of close adaptation to the gums all around, creating no irritation and admitting of restoring the perfect shape of the teeth in the mouth, a great advantage in talking.

*Dr. W. H. Morgan*—Said there was one absolute essential to success in the insertion of artificial crowns, and that was the proper medication and treatment of the root. Success depends more upon the healthy condition of the root than upon the manipulation of the crown. It is well to lay especial stress upon that point, for he had removed beautiful crowns that had been built upon teeth of which the pulp-chamber had never been opened. Of course periostitis had followed. Another point in regard to impacting gold: every angle made in the foil previous to placing it in the cavity requires that much more pressure in compacting. If there are a hundred angles in the pellet it may require a pressure of one pound to flatten it out; if there are a thousand angles it will require ten pounds' pressure. Gold, in the best form, has smooth surfaces ; not crumpled up with a thousand angles to break down. Some one said that a filling should be perfectly solid, but there are cases when to be solidly compact throughout is a disadvantage. Where there is no room for expansion, thermal changes will break down frail edges. Hundreds of teeth are now saved that the fathers would have condemned to the forceps; they are saved by our new methods and new materials. He would use matrices in certain localities, but not everywhere. It was said that the matrix made simple cavities in all cases, but this would not always be an advantage, a compound filling being sometimes easier than a simple one.

*Dr. Allport*—I desire to make an explanation. My friend McKellops said that I annealed my gold and used the mallet, and *therefore* I used cohesive gold. I desire to say that the larger part of the gold that I use in the filling of a cavity is non-cohesive. When I build up outside of a cavity I use, as all dentists do, cohesive gold. But I will explain how I use my gold, so that no one need misunderstand me. Suppose I make a retaining point—which I seldom do, but which those who use cohesive gold are usually obliged to do—no man can fill the retaining point as perfectly with cohesive as with non-cohesive gold. I do it in this way: I take a little pellet and anneal a small point; then I put the non-cohesive portion down into my retaining point and fill it perfectly, leaving the cohesive portion on the surface; then take another piece in the same way, putting the non-cohesive point of the second pellet on the cohesive point of the first, every time. With this little point of cohesion there is no danger of the second piece tumbling off, or rolling about in the cavity. The greatest part of my filling is, therefore, of non-cohesive gold. My friend seems to think that because I use the mallet I must use cohesive gold exclusively. In this he is entirely mistaken, for I introduce the gold, in nearly all my operations, with mallet force. I only use hand instruments and hand pressure in those locations in which it is difficult to make perfect operations with mallet instruments.

*Dr. W. O. Kulp*—This is a mixed discussion, and I am going back to bridge-work again. It is an old saying that the proof of the pudding is in the eating. It is the same with bridge-work. I wear it myself, and speak whereof I know. I had the misfortune to lose a molar and a bicuspid, and tried twenty different plates and appliances. Later on I lost a lower molar on the other side. I could not masticate on one side with my artificial appliance, nor on the other side because of the lost molar. The other molars began to abrade, and the front teeth to push forward and become sore. My attention was directed to what we call a quack advertisement of bridge-work. I concluded to beard the lion in his den. I wanted my jaws separated somewhat, to save the front teeth. I had the bicuspids and molar capped, and a bridge put in to supply the lost teeth. On the other side I had the molar capped, and the inner cusp of the bicuspid taken off. My powers of mastication are fully restored, and I have worn the work for two years with entire satisfaction. When front teeth are abraded, separate the jaws with caps.

*Dr. J. J. R. Patrick*—I hold it man's first duty to know what the material is that he is using. We hear the terms adhesive, and cohesive, and non-cohesive, and these create some confusion. All gold is cohesive if good, where there is nothing to interfere with one molecule coming in absolute contact with another. There are many different names for gold, but they are merely commercial names. One molecule interlocks with another, and they become homogeneous. When adhesive, something intervenes and prevents the unity of molecules. Non-cohesive seems to mean adhesive—brought together, and held together like two pieces of wood glued with mucilage. We have spent much time in discussing the Herbst method. It serves a purpose and teaches us how to do better. I cannot see how we can get cohesive results as well as by the direct blow. If gold was of the same character as butter, it might be done. We must have direct percussion to drive the molecules into each other.

*Dr. Dyer*—Gold is adhesive when absolutely pure; passing it through the lamp makes it pure by destroying any film of extraneous matter, and makes it cohesive also. Adhesiveness means adhering to some foreign body; cohesive is where the particles of gold unite when brought together. Cohesive gold is very soft, but so-called "soft gold" is not as soft as cohesive gold when passed through the lamp.

*Dr. W. B. Ames*—Said that he was an advocate of eclecticism in practice. But he could not see the consistency of putting a soft mass in the bottom and using the mallet to condense the surface. By using non-cohesive gold, by hand or mallet pressure, we get a solid foundation on which to use the electric mallet for finishing the surface. What objection is there to the mallet? Why depend on welding? What difference does it make, provided that it is made solid? When should we use the mallet and when not? In putting gold in inaccessible points, he was in favor of hand-pressure as giving better command of the instrument, whether using cohesive or non-cohesive gold.

*Dr. J. Taft*—Said that Herbst did not stand alone in his method. In 1881, at the International Medical Congress in London, in the dental section, he saw specimens of cavities lined by Dr. Blount in the same manner, with a smooth, pointed instrument. It was like pasting gold upon a smooth surface. The smooth walls were lined with non-cohesive gold in this manner, and the contour finished with cohesive gold; the lining was very perfectly done and was much admired. The advantage claimed was the thorough adaptation to the walls, better than by any other method. No one could doubt, after seeing the specimens and witnessing the operation, that it possesses some advantages over all other methods. The principle of the Herbst Method is that of a lining, all over the walls, of soft foil, introduced with smooth points with a rotary motion. The centre can then be filled up with cohesive gold. As to the use of the matrix—Dr. Daboll says that all young men should use the matrix in every case of approximal fillings. This must be taken with considerable caution, as it is liable to mislead. Dr. Daboll said that the use of the matrix reduces all proximal cavities to simple crown cavities. There are some points, in filling with the matrix, which are not common to crown cavities, as in the adaptation to cervical edges, and to the lateral borders all the way up. Where the matrix comes up square against the cervical border there will be a deficiency at that point, unless the greatest skill is exercised and the very finest instruments used. It requires more skill than is possessed, except by the very few. He would not advise the use of the matrix except where absolutely essential. The matrix will not fit up to curved cervical borders. Dr. Patrick has said no man ought to use a thing that he did not know all about. If that was the case we must all go to school.

*Dr. C. W. Spalding*—Wished to ask one question. Dr. Taft had said *soft foil* and then corrected himself, saying non-cohesive. He would like that point explained.

*Dr. Taft*—The terms soft and non-cohesive are carelessly used as interchangeable. Sometimes gold is quite soft, like butter or cheese, yet it will weld. All depends on the management of the gold in its preparation. Those who have worked gold know that there is a great variety of behavior in gold, as in any other metal. Variety may be given by the manner in which it is treated. Pure gold is always the same in quality, but temporary qualities are given to it by the method of treatment.

*Dr. Brophy*—Said that when gold was submitted to fumes of aqua-ammonia it became non-cohesive. He had no choice in buying one rather than the other. If he wanted it cohesive he annealed it; if he wanted it non-cohesive he placed it in a drawer where it was exposed to the fumes of ammonia. He said that he had had no experience in constructing bridge-work, but he had examined the work of others. It claimed to be something strong and simple, supplying missing teeth without a plate. But what he had seen was anything but satisfactory, in his judgment. For one or two teeth it might serve a very good purpose for a certain time, but for four or five teeth—say the first molar and two bicuspids even—it would soon result in failure. It was not possible to secure absolute adaptation of the teeth. There would sooner or later be a space in which secretions would accumulate and become exceedingly offensive, destroying the adjacent teeth. He would rather be without several teeth than wear such bridge-work as he had seen. In the adaptation of incisor teeth there must be gold visible in bridge-work. Why not have a gold plate? It would serve a better purpose. The bridge-work would, in a few years, result in the loss of the teeth to which it was attached.

*Dr. W. P. Horton*—Said that the present discussion of operative dentistry seemed to cover the whole field of practice. It had been said that the old methods saved as large a proportion of teeth as was saved at the present time. He believed that though the old fathers had but little light, they worked up to the light they had. The introduction of adhesive foil has added to the light, so that hundreds of teeth are saved now to one filled then. Teeth that formerly were doomed to the forceps were now saved. Herbst does not stand alone in his method. Blount gave his instruments a rotary motion, not rapid, but rotating sufficiently to carry the foil to the cervical walls. No man can succeed thoroughly till he has a knowledge of all methods. Combine the various systems with sound judgment. The head, hand, and heart must all be educated in this work.

On motion, the subject of Operative Dentistry—Section IV—was passed.

A communication was received from the Superintendent of Memorials for the Central Woman's Christian Temperance Union, asking the adoption of the following resolutions:

*Resolved.*—That we recognize and commend the excellent work done quietly, but systematically, by the Woman's Christian Temperance Union.

*Resolved.*—That we endorse its movement for temperance education in the public schools; its proclamation against the use of alcoholic stimulants, and the efforts it is making to educate the people in regard to the laws of Hygiene and Heredity.

*Resolved.*—That as a body of practitioners, for the purpose of developing a moral feeling throughout the country, and as having a direct influence for good upon all classes, we will discourage the use of alcoholic stimulants in our practice.

Respectfully submitted.

Dr. C. N. Pierce, moved the adoption of the resolutions.

Dr. A. T. Smith, moved that they be so amended as to include the use of tobacco.

Dr. C. W. Spalding, moved that they be laid on the table.

Dr. W. C. Barrett, seconded the latter motion, saying that he did this, not with any intention of discourtesy, but because it was a matter entirely foreign to the objects of the association —to the practice and science of dentistry.

The motion to lay on the table was carried.

On motion, adjourned to 3 P. M.

THURSDAY AFTERNOON SESSION.

The President in the chair.

On motion of Dr. A. W. Harlan, Surgery was added to Section VI, no special provision having been made heretofore for papers for discussions on that subject.

On motion of Dr. Barrett, Section V was passed, and Section VI called.

Dr. A. W. Harlan, chairman, reported a paper from Dr. J. D. Patterson, entitled "The Catarrhal Nature of Pyorrhœa Alveolaris,"

and one from Dr. Wm. H. Atkinson, on "Pyorrhea and Sponge Grafting," also his own written report as chairman of the Section.

Briefly summarized, Dr. Harlan said that there had been few discoveries in dental pathology, during the past year, of sufficient significance to be placed on record, the most momentous being the researches of Prof. Miller, of Berlin, the vast import of which was not yet appreciated or understood. He had not been able to complete his report on Pyorrhœa Alveolaris, and asked for further time.

It is important that we be able to extend the list of remedies; the most valuable and interesting drug added during the past year was Cocaine. His conclusion, from his own experience, was that Cannabis Indica was vastly more efficacious than any form of Cocaine; that the former would obtund the most sensitive cavity, while painting the gums with the fluid extract renders them absolutely insensible to pain; an exposed pulp was anæsthetized in from five to ten minutes. By saturating the gums, extraction is rendered painless when done with warm forceps dipped in the tincture. He had not had good results from Cannabis Indica in pyorrhœal pockets. The antidotes were the same as for Opium.

Resorcin and Terabin were two new remedies, as yet not generally appreciated by dentists. Resorcin was a valuable substitute for carbolic acid, being neither poisonous nor escharotic, except internally. Terabin—transparent and limpid, and a ready absorbent of oxygen—is stimulating, disinfectant, and antiseptic, and in closed abscess it destroys all foul odor when used as a dressing for the mal-odorous root-canals. It has no obtunding qualities. Its antiseptic merits are undoubted.

*Dr. Davidson*—Said where he had failed with hydrochlorate of cocaine, he had succeeded perfectly with cannabis indica. He described a case of caries of the lower central incisor, running to the margin of the gum and very sensitive. He applied the dam and cannabis indica, and with the engine removed a large portion of dentine without any complaint from the patient. On the first symptom of pain he again applied the remedy, working on another tooth for a few moments, when he returned to the central incisor without warning to the patient, and it was all right again.

*Dr. A. W. Harlan*—Said that he had not been able to obtain as good results from cannabis indica as from cocaine; that the preparations of cannabis indica were very uncertain, and he could get no two supplies alike. One would act beautifully, while from another the results would be *nil.* Even from the samples given out that morning, he could get no results on the tongue. It produced no effect on sensitive dentine, but left a peculiar sensation in the teeth for thirty-six hours. He had had many cases of pyorrhœa that he could not attribute to local causes, but that were probably of constitutional origin, as in patients of rheumatic or gouty diathesis, or suffering from glucosed urea, Bright's disease, etc. In such cases local treatment had been of no avail until the physician had mitigated the disease, when the pyorrhœa was also better. The same was true in the case of ladies with uterine troubles. In these cases there would be pyorrhœa without deposits, and all local treatment vain until the patient was successfully treated by the gynæcologist, when there would be corresponding improvement in the mouth.

Dr. J. D. Patterson then read his paper entitled "The Catarrhal Nature of Pyorrhœa Alveolaris." This essay was an elaborate study of the comparative pathology of Pyorrhœa and Catarrh. He said that Catarrh was distinctively an inflammation of the air passages, distinguished as nasal, pharyngeal, laryngeal, bronchial, etc. That the exudations changed from watery to pus and blood, the serous effusions being irritant and excoriating; that it was exceedingly infectious, adjoining parts being promptly affected. Its further stages were inflammation, destruction of function, formation of crusts, decomposition, and ozœna; the formation of deep and penetrating ulcers producing necrosis, with bony deposits and osseous formations under the crusts. These catarrhal phenomena, affecting the cavities of the upper jaw, strongly resemble those of pyorrhœa, in the inflammation of the gums, exudations of serum, pus, and blood, the destruction of the ligaments of attachment, the deepening pockets, the calcareous deposits, and the absorption or necrosis of the septum and alveolus. Careful attention was directed to the similar pathology :

1st. In the similar affection of the mucous membrane.

2d. The identical character of the effusions—purulent serum, pus, and blood.

3d. Its infectious character, affecting adjoining teeth in pyorrhœa as it does adjacent parts in catarrh.

4th. The similar burrowing of pus.

5th. The destruction of periosteum and bone.

6th. The similar deposits—that of pyorrhœa being familiar, the osseous deposits of catarrh being also fully described by authors, as in Ziemsen's Medical Encyclopedia, where the chalky deposits and bony concretions of catarrh are described.

These points of similarity lead to the conclusion that the two affections are similar in origin and in nature, catarrh being a disease of the air passages, and all tracts to the lungs being liable to contamination, while pyorrhœa is the result of local contamination from nasal drippings, or from poisonous sputa lodging around the teeth, or, in case of mouth-breathers, originating in the gums as readily as in the nasal passages. Dr. Patterson said that he had made a special study of twenty-four cases. In many instances, where catarrh was denied, it was found present, and his observations had confirmed his theory in every case. The patient may be ignorant of and deny the presence of catarrh, even when hypertrophic and fetid symptoms are readily recognized by others.

*Dr. Thompson*—Said he had had a case of pyorrhœa in a patient whose habits were not cleanly, and whose teeth were very bad. He had also a very offensive case of catarrh, but had never had any treatment. He prescribed preliminary treatment for the catarrh, with salt water douche, etc. The improvement in the catarrh resulted in marked improvement of the pyorrhœa also. The blending in pyorrhœa of most of the symptoms of catarrh suggested that when pyorrhœa was present there was also nasal catarrh, and he found this invariably confirmed by the physician. He had investigated, after the suggestion of its renal origin, but could not trace it to that source. He said that Dr. J. G. Templeton claimed wonderful results from the application of copper sulphate—blue stone—in the pockets once a week. That he had considerable experience in the use of cocaine, meeting with some successes and some failures—success in perhaps sixty or seventy per cent—but that it was impracticable in many cases, requiring too much time to produce its results. Neither he nor his patients could afford to wait twenty minutes, and then twenty more. By taking full time the desired result could doubtless always be secured ; it was reliable, but the time required made it impracticable.

*Dr. King*—Said that he had some experience and some success in the treatment of pyorrhœa. Having kept a record of the history of a large number of cases, with the constitutional history of each patient, his experience failed to confirm the theory that the disease was due to an impoverished condition of the blood, at least fifty per cent being people in good health, with robust, vigorous constitutions. He considered that calcareous or ceruminal deposits were the immediate cause of the disease, though there was, of course, something back to cause the deposits. If this was not so, why was it so essential to remove all deposits? Why insist on keeping the teeth free from subsequent deposits to prevent the return of the disease? In cases where no salivary deposits are present, it will always be found, on tracing the case back, that it had been the initial trouble, though the system had so changed, more recently, that the deposits were not continued, but the track was there.

*Dr. Ingersoll*—Wished to correct a statement which had recently appeared in the journals, as quoted from Dr. Rawl's paper on Pyorrhœa, where he (Ingersoll) was represented as saying that the gathering of salivary calculus was the cause; it was never the cause, but uniformly the result.

*Dr. Friedrichs*—Wished to inquire whether there were any recognized cases of calculus without pyorrhœa? He had had one case where there were considerable deposits which the patient would not allow touched, and which he said had been unchanged for twenty-five years. The gums were of healthy, natural color; no pus, no inflammation; the alveolus very thin.

(TO BE CONTINUED.)

